# High extinction ratio electromagnetically induced transparency analogue based on the radiation suppression of dark modes

**DOI:** 10.1038/s41598-017-11920-8

**Published:** 2017-09-12

**Authors:** JingYa Xie, Xi Zhu, XiaoFei Zang, QingQing Cheng, YangYang Ye, YiMing Zhu

**Affiliations:** 1 Terahertz Technology Innovation Research Institute, Shanghai Key Lab of Modern Optical System, and Engineering Research Center of Optical Instrument and System, Ministry of Education, University of Shanghai for Science and Technology, No. 516 JunGong Road, Shanghai, 200093 China; 2 Terahertz Science Cooperative Innovation Center, Chengdu, 610054 China

## Abstract

A high extinction ratio (ER) electromagnetically induced transparency (EIT) analogue based on single-layer metamaterial is designed and experimentally demonstrated in this paper. This design involves four mirror-like symmetrically coupled split ring resonators (SRRs) that exhibit a bright-dark-dark-bright mode configuration. The EIT-like effect is realized by coupling between the bright resonators and dark resonators. The high ER feature is achieved from the suppression of radiative losses, due to opposite directions of electric and magnetic dipoles of two dark modes in the unit cell. Classical coupled resonator model is used to theoretically analyze the device transmission performances and to characterize parameter influence of the ER. Both numerical simulation and experiment results demonstrate that the ER of this device can reach more than 21 dB, which is 11 dB higher than that of conventional bright-dark coupling SRR arrangement. Finally, the potential multi-channel sensing utility of this device is demonstrated to show the importance of high ER feature.

## Introduction

Electromagnetically induced transparency (EIT) is originally observed in atomic physics and arises due to quantum interference effect, resulting in a narrow transmission window inside an absorption band^1^. This concept was later extended to classical oscillator systems, such as mechanical spring-mass configurations^2^, RLC electric circuits^3^, coupled optical micro-resonators^4, 5^, and waveguides side coupled to a resonator^6, 7^. In the field of metamaterials, the transparent and highly dispersive nature of EIT-like effect offers a potential solution towards low-loss and high-quality-factor resonances, which are critical for realizing low-loss slow-light devices^8^, sensors^9^, filters^10^, photoluminescence^11^ and enhancing nonlinear interactions^12^. The classical analogue of EIT in metamaterials relies on the coupling between a broadband ‘bright’-mode resonator, which is accessible from free space, and a narrowband ‘dark’ mode resonator, which is less-accessible from free space^13^. Due to low radiative loss of the dark mode, the EIT-like resonance can be very sharp, resulting in complete transmission^14^ or reflection^15^.

The EIT-like effect has been realized in a variety of metamaterial configurations, such as nanoring dimer, metal strips and coupled split ring resonators^16–21^. Active control and tuning the transparency window as well as its slow slight properties have also been explored in the past few years^5, 22, 23^. An appealing scheme was to use two polarization dependent split-ring resonators (SRRs) work as bright and dark modes respectively^24, 25^. However, the extinction ratio (ER) of conventional bright-dark SRR resonance is quite low due to its high radiative losses^25, 26^. Since high ER resonance is of particular importance for the ultrasensitive resonance sensing or frequency-selective ability in filter applications^27^, it is necessary to further suppress the radiation loss of bright or dark mode and increase ER in designing such devices.

In this work, we propose and demonstrate a mirror-like symmetrical unit cell of metamaterial to generate an EIT analogue with high extinction ratio. The unit cell consists of four SRRs to form the bright-dark-dark-bright modes configuration. Two bright resonators can be strongly excited by the incident wave, while two dark resonators can only be excited through near field coupling with bright SRRs. Compared to the conventional bright-dark coupling of two SRRs, the symmetrical arrangement of two dark modes induces highly suppression of their own electric and magnetic dipole radiation losses, and hence extraordinarily high ER transmission. A theoretical analysis using the coupled-resonator model shows the transmission characteristics, and numerical simulation is adapted to analyze interaction between the four elements in the metamaterial unit cell. The transmission performance is measured for fabricated devices and 11 dB improvement of ER is obtained, which verifies our design. Finally, we demonstrate the sensing ability of our structure and compare the results with conventional bright-dark structure, to further show the potential utility of this multi-channel sensor and the importance of high ER feature.

## Results and Discussions

**Figure 1:**
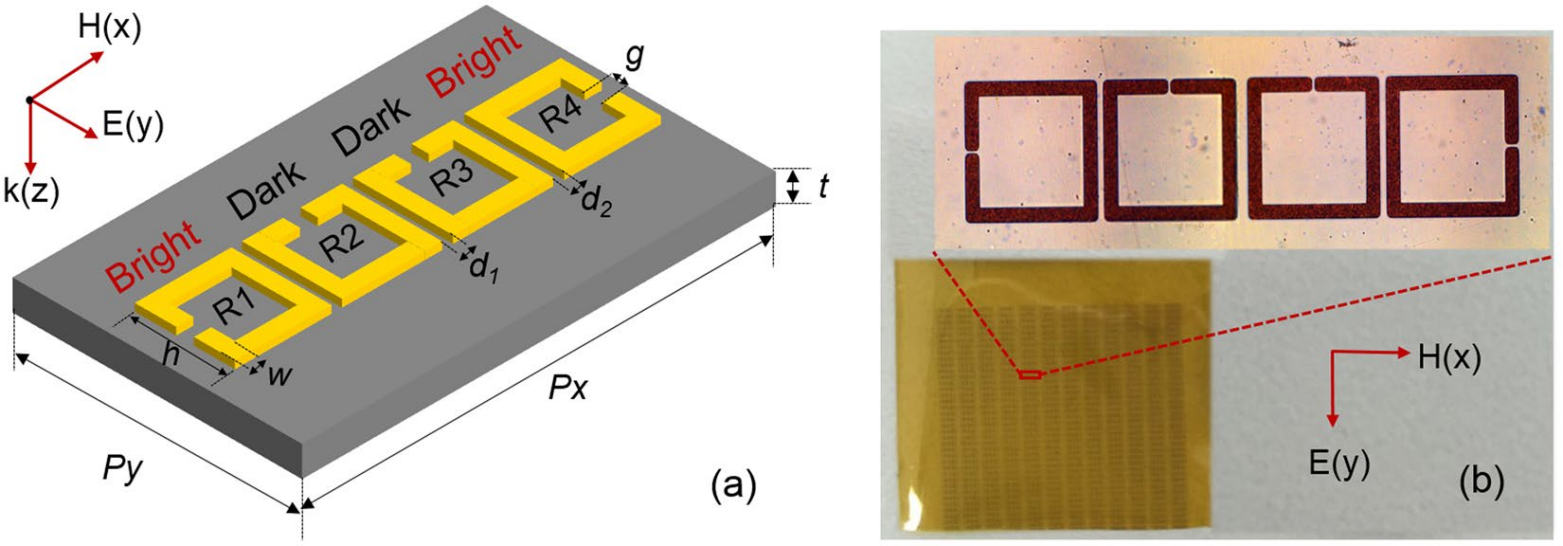
The schematic illustration of the mirror-like symmetrical metamaterial unit cell. (**b**) Image of the fabricated sample.

Figure 1(a) shows the schematic illustration of our proposed symmetrically coupled bright-dark-dark-bright metamaterial unit cell that consists of four SRRs, such unit cell appears periodically to form a metamaterial structure of Fig. 1(b). In each unit cell, the two SRRs with the split gap aligned parallel to the electric field of incident wave are termed as bright resonators (R1 and R4), since their fundamental LC resonances can be excited. On the contrary, the other two orthogonally twisted resonators (R2 and R3) are called dark resonators, as their fundamental LC resonance is less accessible with the same polarization. Therefore, the unit cell is composed of the bright-dark-dark-bright mode resonators, which are coupled through their electromagnetic near-fields. The interference between bright and dark modes form a typical EIT-like system, and the near-field coupling between dark modes results in the changing of resonance shape. More importantly, the symmetrical structure generates electric and magnetic dipoles in opposite directions to make further suppression of dark mode radiative losses. The geometric parameters of optimized unit cell are illustrated as follows: all the SRRs are square and the length of their sides are *h* = 216 µm, the width of metallic arm *w* = 18 µm, the size of all the gaps is *g* = 9 µm, the distance between R2 and R3 is *d*_1_ = 20 µm. The distance between bright and dark resonators are all set to *d*_2_ = 16 µm. To form a metamaterial structure, the periods in the x direction is *P*_*x*_ = 1250 µm, and the periods in the y direction is *P*_*y*_ = 400 µm. The traditional bright-dark mode EIT-like metamaterial is also designed and fabricated to compare with our device, which consists of two SRRs with the same above dimensions. It is necessary to note that the ER of bright-dark SRRs varies little with the changing of the geometric size, as shown in numerical simulation results in supplementary section 1. Its periods in x and y direction are 625 µm and 400 µm, respectively. The total sample array has a footprint of 15 mm × 15 mm = 225 mm^2^.

To explore the physical origin of symmetrically near-field coupled bright-dark-dark-bright metamaterial, the widely used coupled resonator model is adapted to analyze the interaction between the four SRR elements in the unit cell^28, 29^. The SRRs R1 and R4 of bright modes are represented by resonators 1 and 4, which can strongly couple with the incident light *E(t)*. In our model, ${E}_{1}(t)={E}_{4}(t)={E}_{0}{e}^{i\omega t}$ due to the fact that different part of the planar metamaterial under normal incidence will suffer the same applied electromagnetic field. The SRRs R2 and R3 of dark modes are represented by resonators 2 and 3, which can be excited only through near-field coupling with resonators 1 and 4. Next-nearest-neighbor coupling is neglectful, thus not included in our model. In order to gain the EIT-like spectral profile, the resonance frequency of all resonators are equal and denoted as ${\omega }_{0}$. The mode energy amplitudes of these four resonators *a*_1_, *a*_2_, *a*_3_ and *a*_4_ satisfy the coupled differential equations as bellow:

1 $$\{\begin{array}{c}\frac{d{a}_{1}}{dt}=i{\omega }_{0}{a}_{1}-{\gamma }_{1}{a}_{1}-i{k}_{12}{a}_{2}+ig{E}_{0}{e}^{i\omega t}\\ \frac{d{a}_{2}}{dt}=i{\omega }_{0}{a}_{2}-{\gamma }_{2}{a}_{2}-i{k}_{12}{a}_{1}-i{k}_{23}{a}_{3}\\ \frac{d{a}_{3}}{dt}=i{\omega }_{0}{a}_{3}-{\gamma }_{3}{a}_{3}-i{k}_{23}{a}_{2}-i{k}_{34}{a}_{4}\\ \frac{d{a}_{4}}{dt}=i{\omega }_{0}{a}_{4}-{\gamma }_{4}{a}_{4}-i{k}_{34}{a}_{3}+ig{E}_{0}{e}^{i\omega t}\end{array}$$

where ${\gamma }_{1}$, ${\gamma }_{2}$, ${\gamma }_{3}$ and ${\gamma }_{4}$ are the damping rates of resonators. *k*_12_, *k*_23_, and *k*_34_ are the near field coupling coefficients between neighbouring resonators. *g* represents a parameter indicating the coupling extent between the radiative mode and the incident electromagnetic wave. Based on the mirror-like symmetrical structure, we have ${\gamma }_{1}={\gamma }_{4}$, ${\gamma }_{2}={\gamma }_{3}$, ${k}_{12}={k}_{34}$, ${a}_{1}(t)={a}_{4}(t)$. If resonators 2 and 3 work as symmetric case, ${a}_{2}(t)={a}_{3}(t)$ and *k*_23_ is positive. Otherwise, ${a}_{2}(t)=-{a}_{3}(t)$ and *k*_23_ is negative. Since the dark mode resonators do not couple with the incident light, we only give solutions for ${a}_{1}(t)$ and ${a}_{4}(t)$. Suppose they have the following time-harmonic form:

2 $${a}_{1}(t)={a}_{4}(t)=N{e}^{-i\omega t}$$

where *N* is a constant. From Eq. (1), the mode energy amplitude of resonators 1 and 4 can be calculated as:

3 $${a}_{1}(t)={a}_{4}(t)=\frac{ig{E}_{0}({\omega }_{0}-\omega +i{\gamma }_{2}-|{k}_{23}|)}{[(i\omega -i{\omega }_{0}+{\gamma }_{1})({\omega }_{0}-\omega +i{\gamma }_{2}-|{k}_{23}|)+i{k}_{12}^{2}]}{e}^{i\omega t}$$

**Figure 2:**
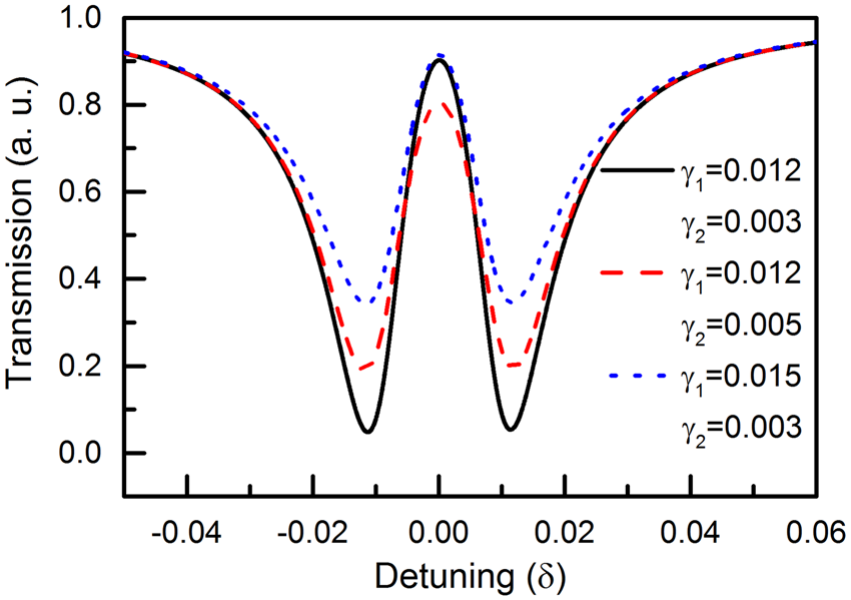
The transmission power versus the detuning frequency at different damping rates.

It is worth mentioning that the form of amplitude *a* is different from the results obtained in one bright-dark mode coupling system^12^, because in our model the dark oscillator can also induce the near-field coupling which affects the final transmission. The foregoing energy flow *P*(*ω*) as a function of frequency is obtained by: $P(\omega )=1-({|{a}_{1}(\omega )|}^{2}+{|{a}_{4}(\omega )|}^{2})$ ^26^, which is shown in Fig. 2. By analyzing this transmission spectrum, we see that the main parameters affecting the extinction ratio (the ratio of maximum to minimum power, defined as 10 log10 (*P*_high_/*P*_low_)) are the damping rates ${\gamma }_{1}$ and ${\gamma }_{2}$, which is shown in Fig. 2 as three curves: the black-solid curve shows the reference transmission spectrum with $g{E}_{0}=0.01$, ${\gamma }_{1}=0.012$, ${\gamma }_{2}=0.003$, ${k}_{12}=0.01$, ${k}_{23}=0.0001$, the blue-doted curve shows the result with ${\gamma }_{1}$ increased to 0.015, and when ${\gamma }_{2}$ is increased to 0.005, the transmission spectrum is shown by red-dash curve. The results show that ER is increased with lower damping rates ${\gamma }_{1}$ and ${\gamma }_{2}$.

The damping rates $\gamma$ (indicating the losses for resonators) are affected by two factors: radiation losses and dissipation losses. Radiation losses are resulted from electromagnetic energies scattered by metamaterial elements away from the incident wave, while dissipation losses arise from intrinsic ohmic losses of materials. It has been clearly shown that radiation losses dominate and far exceed dissipation losses, even in the optical regime, where the ohmic losses are high^30^. The bright-mode resonances are strongly coupled to free space, leading to very high radiation losses and low Q factors. Potentially the most appealing approach to suppress their radiation losses is to introduce dark-mode coupled SRRs, which results in the EIT-like transmission^31, 32^. Hence, our device leverages the radiation loss suppression of bright-dark coupled SRRs, and further suppress the less-radiation losses of the two dark modes using the symmetrical arrangement.

**Figure 3:**
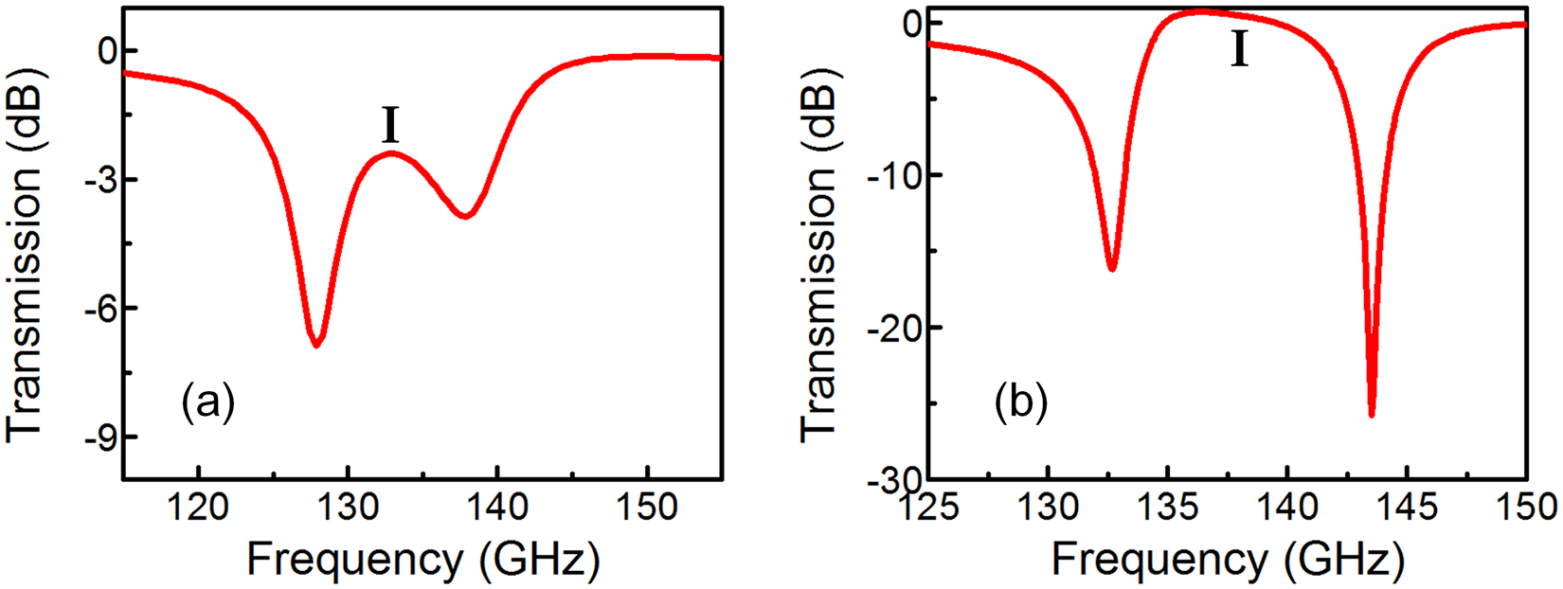
Simulated transmission spectra of (**a**) bright-dark SRRs and (**b**) bright-dark-dark-bright SRRs with the same dimensions.

To further verify our design, we compare the numerical calculation transmission response of bright-dark-dark-bright mode with the bright-dark mode. Figure 3(a) and (b) show the simulated transmission of the two designs with normally y-polarized irradiation. When the resonant frequency of this sub-radiative mode coincides with that of the radiative mode, a sharp and narrow transparency peak (denoted by “I”) appears in the original transmission-forbidden band, resembling the spectral line shape of a typical EIT-like effect in atomic systems. It is seen clearly that by introducing the symmetrical arrangement of the unit cell and forming a bright-dark-dark-bright mode, a higher ER is achieved.

**Figure 4:**
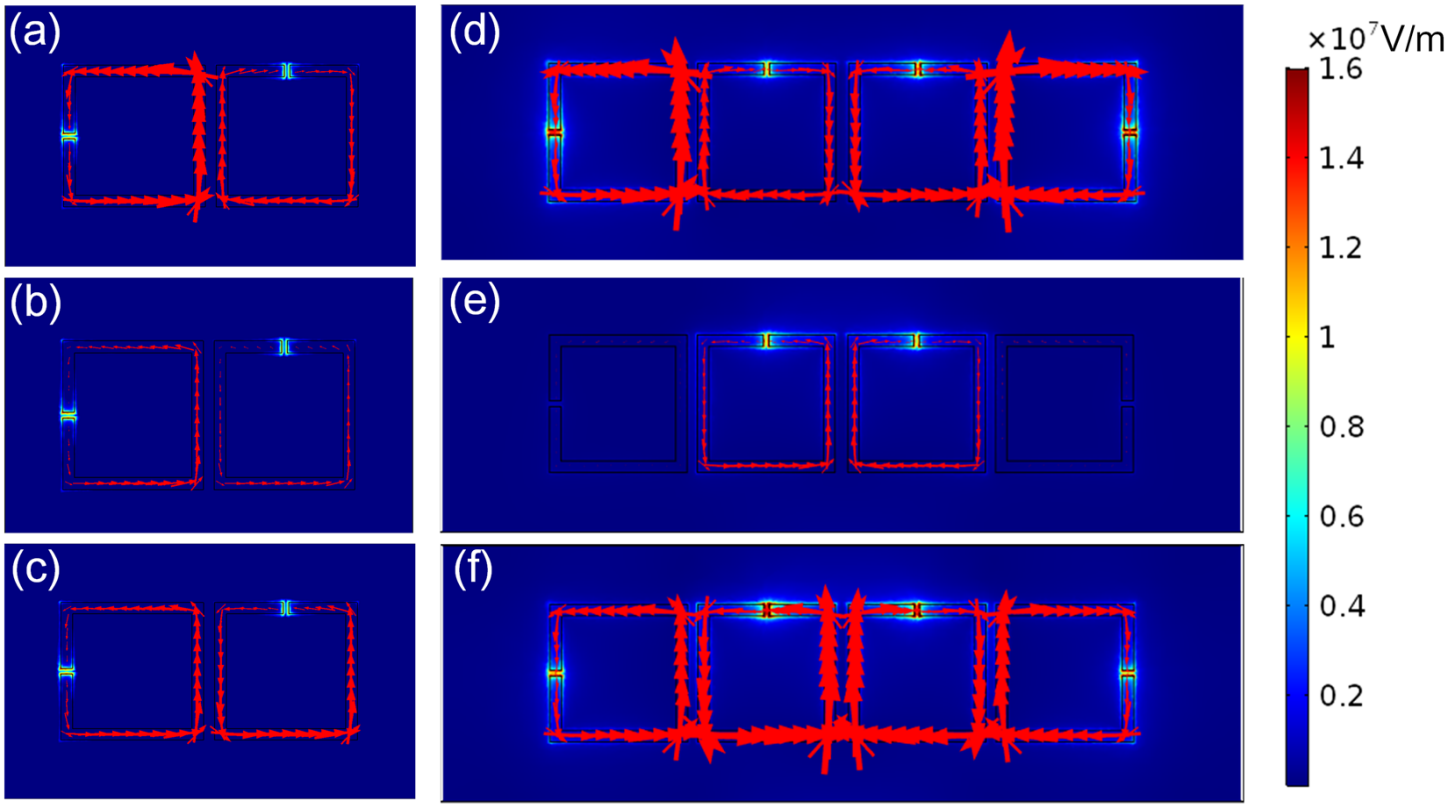
Surface current and electric field distributions of (**a**–**c**) bright-dark SRRs and (**d**–**f**) bright-dark-dark-bright SRRs. All simulations are for three resonant response: the lower asymmetric resonance, the transparency peak, and the symmetric higher resonance. The red arrows indicate the induced surface current density, and the color represents the electric field norm.

To reveal the underlying mechanism of increasing ER and near field coupling of SRRs, we calculated the induced surface current density at the frequency of transparent peaks and dips, as shown in Fig. 4. The direction and size of the red arrows indicate the direction and relative value of the surface current density. One SRR can be viewed as an equivalent inductance-capacitance (LC) circuit, in which the metal ring is regarded as a magnetic loop, and the slit of the ring is a capacitor. In the bright-dark coupled system, the LC resonance mode originates from the resonant electric currents oscillating around the bright resonator, which is induced by the incident wave. Then, the LC mode in the dark SRR is excited by the bright SRR through near-field inductive coupling. With such circular currents in both SRRs, they would couple to each other through their self-consistent electromagnetic field, leading to resonance mode hybridization, which is reflected in clear splitting of the fundamental resonances. The induced surface current densities of two splitting modes oscillate out of phase at the lower asymmetric resonance frequency and in phase at the symmetric higher resonance frequency^24, 33^.

According to classical electrodynamics theory, the equivalent radiation structure of one SRR can be viewed as an electric dipole in the split gap and a magnetic dipole in the loop^34^, with the former much stronger than the latter^35^. In the bright-dark-dark-bright structure shown as Fig. 4(d–f), the currents in the neighbouring dark mode SRRs are in opposite directions (one is clockwise and the other is counter clockwise). As a result, the two dark mode SRRs form opposite electric dipoles (in x and –x direction respectively) and magnetic dipoles (in z and –z direction respectively) shown in supplementary section 2, which lead to lower radiation loss, lower damping rates and thus higher ER transmission. On the other hand, the opposite currents in the two bright mode SRRs form an electronic dipole with the same direction (both in -y direction).

The electric field distributions at the respective transparent peaks and dips are also shown in Fig. 4. The color map shows the relative local electric field amplitude. The incident plane wave drives circulating surface currents in the inductive loops, resulting in charge accumulation at the capacitive split gaps, thus enhancing the electric field in the vicinity of gaps. In the transparent peaks, strong electric field is induced in the dark resonator split gaps (Fig. 4(b) and (e)), and a weak surface current is developed across the bright resonators. The suppression of bright mode electric field in Fig. 4(e) is more significant than that in Fig. 4(b), corresponding to a higher transparency peak (denoted by “I”) in Fig. 3(b). The enhancement of electric field and excitation of LC mode of transmission dips in bright-dark-dark-bright mode is much larger than bright-dark mode, leading to a higher energy absorption in the resonances, which shows good consistency with high ER characteristics.

**Figure 5:**
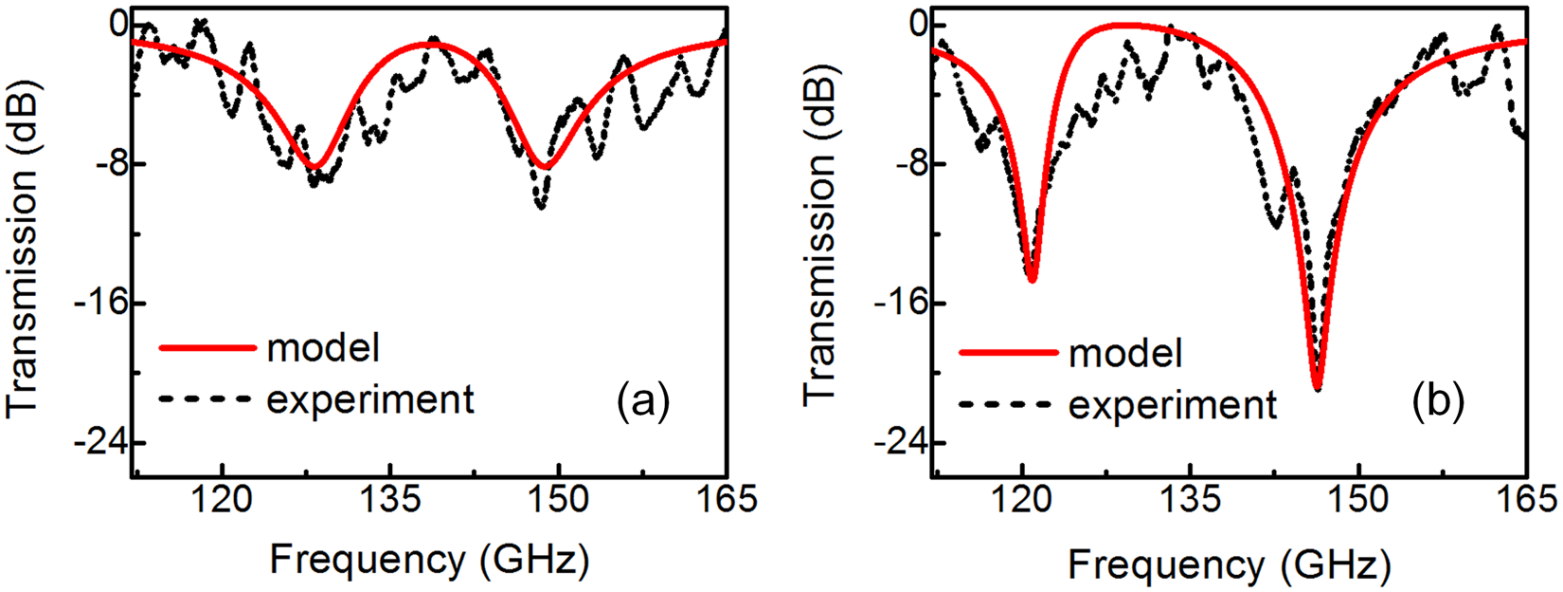
Measured transmission spectra of (**a**) bright-dark SRRs and (**b**) bright-dark-dark-bright SRRs.

In the experiment, we characterize the electromagnetic responses of both bright-dark mode and our bright-dark-dark-bright mode coupling. The transmission spectrum of the bright-dark mode is shown in Fig. 5(a) by the black-dash line. The fundamental LC resonance has the mode splitting and the split resonances appear at 129 GHz and 148.2 GHz. The ERs of transmission dips are measured to be around 9 dB and 10 dB, which shows the basic bright-dark mode coupling characteristic of the resonance. On the other hand, the transmission spectrum of symmetrically coupled bright-dark-dark-bright metamaterial is measured and shown in Fig. 5(b) by the black-dash curve, in which the split resonance is observed at 120.7 GHz and 146.4 GHz. More importantly, the symmetrical structure with the involvement of dark modes damping rate suppression shows significantly enhancement of transmission ER, which are measured to be 15 dB and 21 dB. Furthermore, we fit the experimental transmission spectra in Fig. 5 according to equation (3) and present the results by red-solid curves in the same figure for comparison and verification. It can be seen that the experimental curves are reproduced well by the fitted results. The fitting values of Fig. 5(a) are γ_1_ = 53.25 GHz, γ_2_ = 32.5 GHz, κ_12_ = 55.89 GHz, respectively. γ_1_ is larger than γ_2_, corresponding to the high radiation loss of bright modes. The fitting values of Fig. 5(b) are γ_1_ = 71 GHz, γ_2_ = 0.6 GHz, κ_12_ = 74.52 GHz, and κ_23_ = 55 GHz, respectively. Comparing the two designs, the change of γ_1_ is relatively small, while γ_2_ (representing the damping rate between the dark mode SRRs) is significantly reduced.

**Figure 6:**
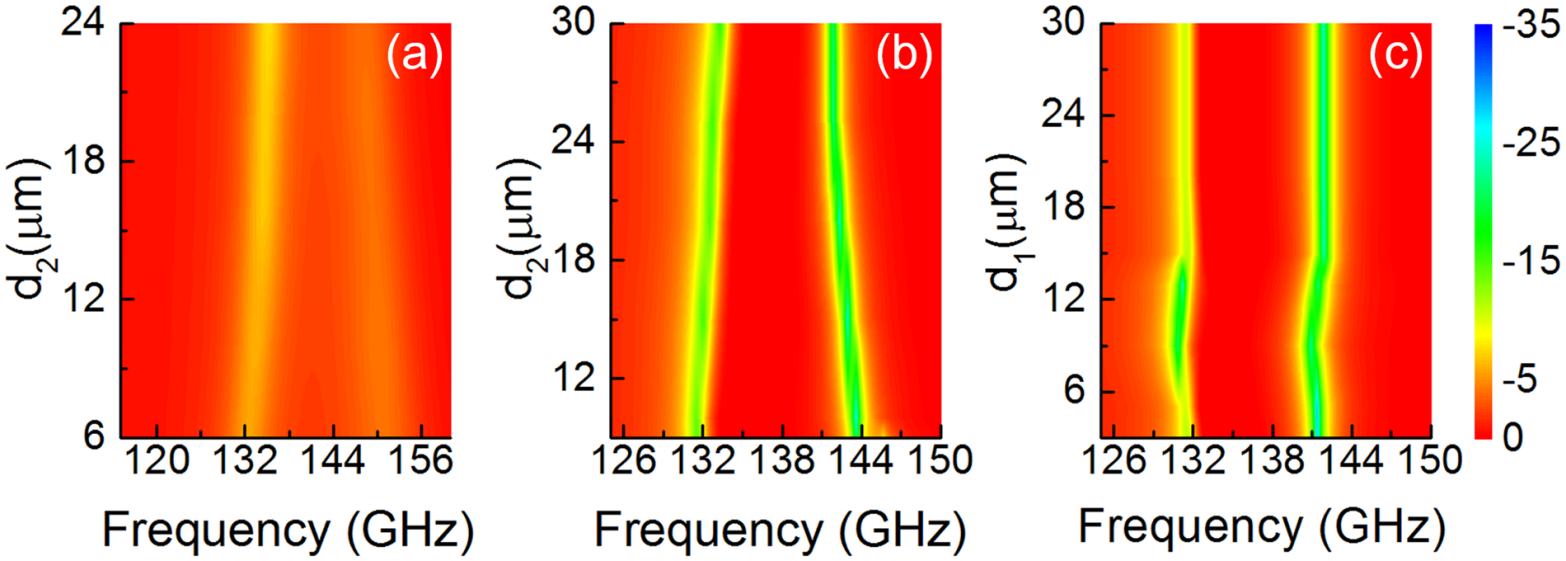
(Color online) Simulated transmission of (**a**) bright-dark SRRs with *d*_2_ changes from 6 to 24 μm, bright-dark-dark-bright SRRs with (**b**) *d*_2_ changes from 10 to 30 μm and (**c**) *d*_1_ changes from 3 to 30 μm.

Comparing the measured results in Fig. 5 with the simulated results in Fig. 3, there are two nonnegligible difference: the mode splitting effect and the position of the transparency peak I. This is most likely due to the fabrication deviation of sizes and distances of the SRRs. In order to explore the effects of the geometrical parameters on the transmission performance, numerous simulations are performed on various parameters setups with different *d*_1_ and *d*_2_, as shown in Fig. 6. In Fig. 6(a), *d*_2_ of bright-dark mode scenario is increased from 6 μm to 24 μm, and all the other geometrical parameters are set to be identical to the experimental sample design. The results show the spectral position of the transparency peak in the EIT like effect remains steady, while the mode splitting increases with the distance *d*_2_ gradually reducing, corresponding to the resonance positions move away from each other. Similarly, the result of bright-dark-dark-bright metamaterial is shown in Fig. 6(b), where *d*_2_ is increased from 10 μm to 30 μm. It is shown that *d*_2_ plays a similar role in mode splitting. On the other hand, while *d*_1_ casts little effect on mode splitting, it shows great influence on ER symmetry of two resonances, which is shown in Fig. 6(c) where *d*_1_ increased from 3 to 30 μm. Such results help explain the resonant frequencies differences between the numerical simulation and experimental results, which can be compensated in design.

**Figure 7:**
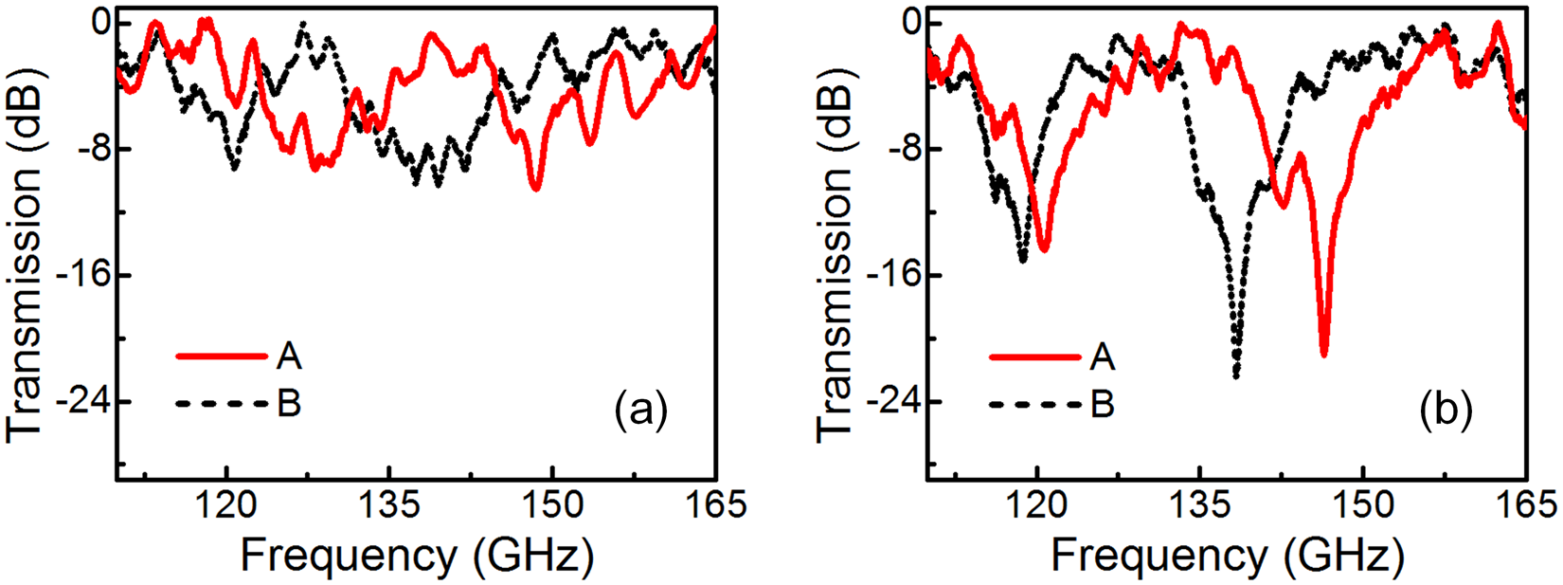
Measured resonance shift of (**a**) bright-dark SRRs and (**b**) bright-dark-dark-bright SRRs. Curve A and B are transmission spectra of samples without overlayer and with overlayer.

One of the potential application of our designed device is to be employed as a thin-film multi-channel sensor, for its resonant frequency is highly dependent on the dielectric constant of the surrounding environment. We then compare the sensing ability of the devices by measuring the spectra for both bright-dark SRRs and bright-dark-dark-bright SRRs. Experimental results are shown in Fig. 7(a) and (b), both metamaterial samples are coated by ~50 μm thick 4-aminobenzoic acid with a relative permittivity ~2.07^36^. As shown by the black-dash curve in Fig. 7(a), the presence of the overlayer causes two dip resonances to shift to lower frequencies of ~120 GHz and ~139 GHz, respectively. The red-shift of resonances is due to the increase in effective capacitance of the split gap. A clear shift of two dip resonances of bright-dark-dark-bright structure is observed at 118.5 GHz and 138.3 GHz respectively, as shown in Fig. 7(b). The sensing performance of metamaterial can be evaluated by a practical concept of figure of merit (*FOM*) as below^37^,

4 $$FOM=\frac{m(\mu m/RIU)}{FWHM(\mu m)}$$

In case of Fig. 7, the $FOM\approx 2.26$, which is close to the value of the nanotubes (*FOM* = 2.4) and SRRs with an asymmetrically coupled resonance (*FOM* = 2.86)^37, 38^. It is worth mentioning that the *FOM* of our proposed EIT-like metamaterial can be further maximized by increasing the separation distance *d*_1_ because the quality factor of the transparency window (corresponding to less mode splitting) grows with *d*_1_, as already demonstrated in Fig. 6. Benefiting from the higher extinction ratio feature, the spectrum shift of our designed structure is clearer to observe and less affected by the power fluctuation. Therefore, such bright-dark-dark-bright structure metamaterial with a higher ER is more suitable to be designed and used as a thin-film sensing device.

In summary, we proposed and demonstrated a symmetrically coupled bright-dark-dark-bright metamaterial to generate an EIT analogue with high extinction ratio. A planar structure composed of four SRRs in each unit cell is used to verify the transmission characteristic. The device is analyzed theoretically with coupled-resonator model, and then supported by numerical simulation that shows the interaction between the four SRR elements. By suppressing the radiation losses of dark modes, the extinction ratio could reach more than 21 dB, which is much higher than conventional bright-dark mode coupling due to the cancelling of electric and magnetic dipoles. Finally, a sensing application is demonstrated and the results show that the spectrum shift of higher extinction ratio EIT-like device we proposed is less affected by the power fluctuation, which indicates good potential in sensing technique for practical applications in environmental, chemical, and biological diagnostics.

## Methods

### Numerical simulation

The transmission spectra, induced surface current density and electric-field distributions are simulated by solving Maxwell’s equations based on the finite element method (FEM). Periodic boundary conditions were used in the x and y directions for simulating an infinite array and port boundary conditions are applied on the top and bottom. The permittivity of the PI substrate was ɛ_pi_ = 3.2 + 0.01i and the electrical conductivity of gold is $4.561\times {10}^{7}S/m$.

### Fabrication and Testing system

The metamaterial sample was fabricated using a surface micromachining process on a polyimide (PI) substrate with thickness of 25 µm. A 100 nm-thick gold (Au) with 10 nm of titanium (Ti) film was E-beam evaporated to create the metal film on the substrate. Lift-off of the photoresist was achieved by rinsing in acetone for several minutes and the metal SRRs were formed on the surface of the polyimide. The transmission characterizes of the sample was measured by using an Agilent N5227A PNA network analyzer and a set of WR-6.5 110–170 GHz VNA extenders. The frequency measurement precision is set to 2 MHz. The polarization of the incident electric field is aligned parallel to the gap-bearing arm of bright SRRs.

## Electronic supplementary material

Supplementary Materials for High extinction ratio electromagnetically induced transparency analogue based on the radiation suppression of dark modes
