# Supplementary material for: High extinction ratio electromagnetically induced transparency analogue based on the radiation suppression of dark modes
Source: Sci Rep. 2017 Sep 12;7:11291. doi: 10.1038/s41598-017-11920-8 (PMC5595855; doi:10.1038/s41598-017-11920-8)
Supplement: Supplementary file 1 — Supplementary Materials for High extinction ratio electromagnetically induced transparency analogue based on the radiation suppression of dark modes [file 41598_2017_11920_MOESM1_ESM.doc]

Supplementary Materials for

**High extinction ratio electromagnetically induced transparency analogue based on the radiation suppression of dark modes**

JingYa Xie^1,2^, Xi Zhu^1^, XiaoFei Zang^1,2^, QingQing Cheng^1,2^, YangYang Ye^1^, and YiMing Zhu^1,2*^

*^1^ Terahertz Technology Innovation Research Institute, Shanghai Key Lab of Modern Optical System, and Engineering Research Center of Optical Instrument and System, Ministry of Education, University of Shanghai for Science and Technology, No.516 JunGong Road, Shanghai 200093, China.*

*^2^ Terahertz Science Cooperative Innovation Center, Chengdu 610054, China*

**Corresponding author: ymzhu@usst.edu.cn*

**Supplementary Section 1. Transmission variation of conventional bright-dark configuration for different dimensions**


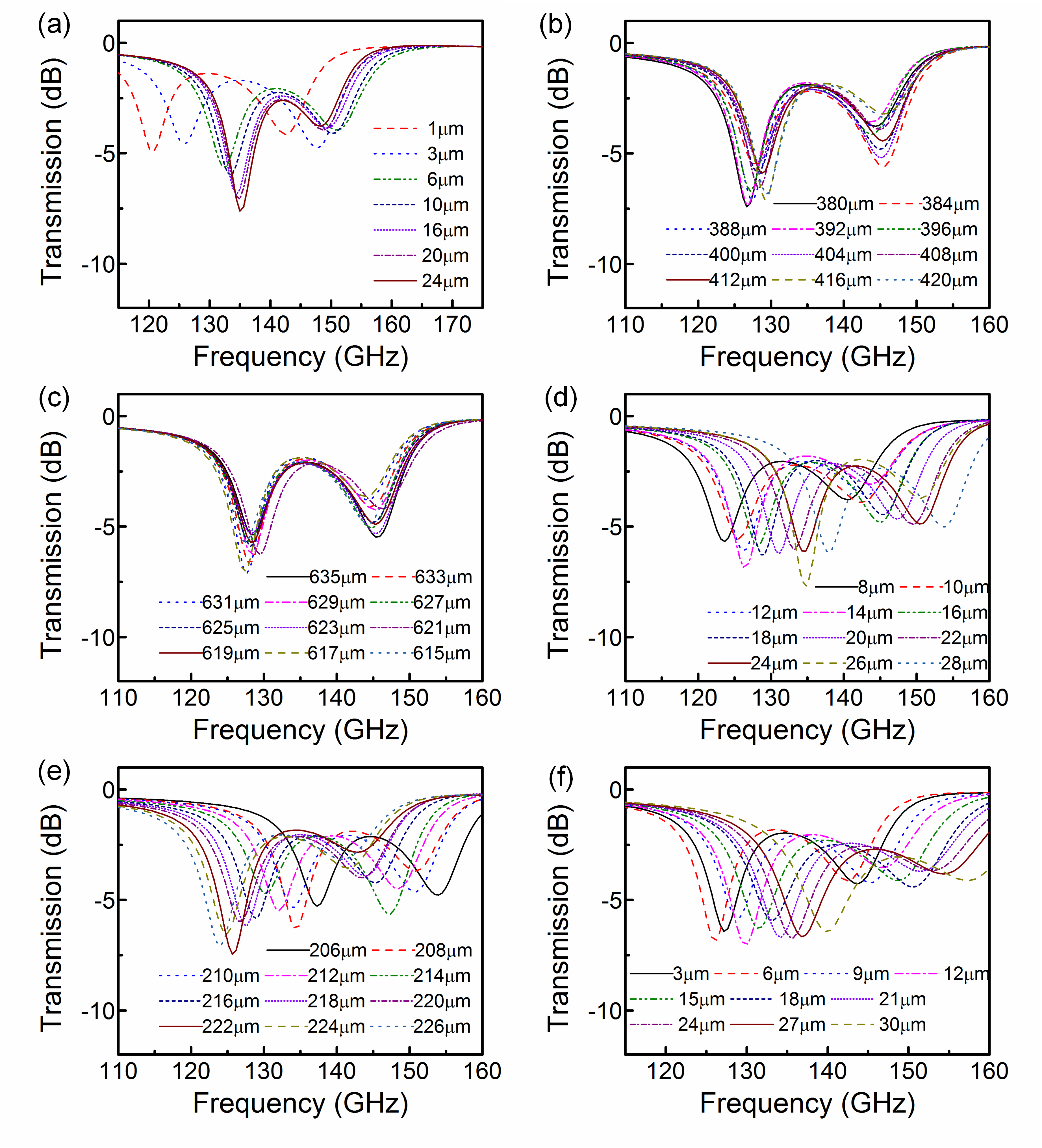


Fig. S1 The transmission variation of bright-dark configuration for different dimensions of (a) *d_2_*, (b) *P_y_*, (c) *P_x_*, (d) *w*, (e) *h*, and (f) *g*.

Figure S1 shows the transmission variation of conventional bright-dark configuration for different dimensions of: (a) *d_2_* (the distance between SRR R_1_ and R_2_), (b) *P_y_* (the periods in the y direction), (c) *P_x_* (the periods in the x direction), (d) *w* (the width of metallic arm), (e) *h* (the length of SRR sides), and (f) *g* (the size of all the gaps).

**Supplementary Section 2. The equivalent dipole model of bright-dark and bright-dark-dark-bright SRRs**


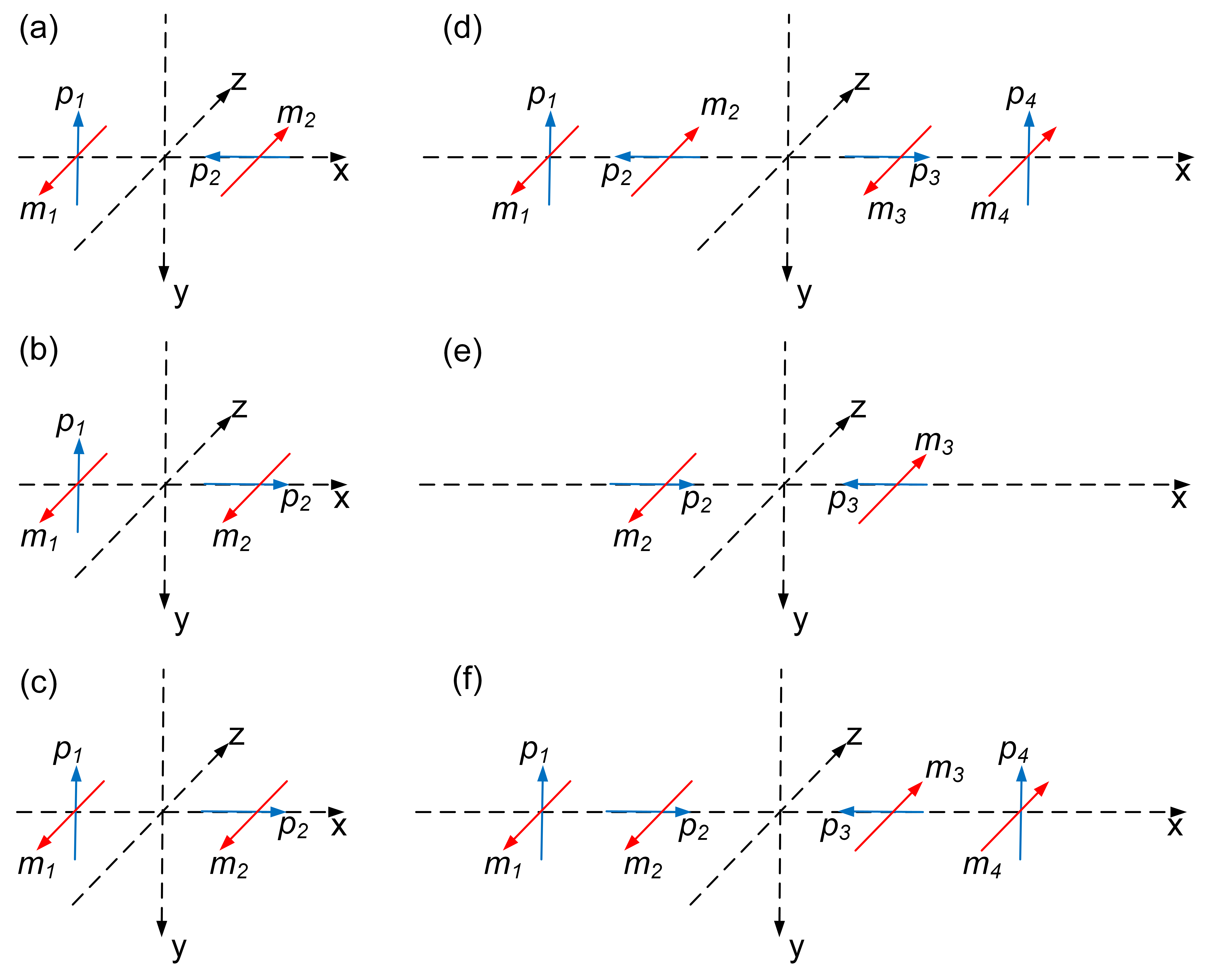


Fig. S2 The equivalent dipole model of (a-c) bright-dark SRRs and (d-f) bright-dark-dark-bright SRRs for three resonant response: the lower asymmetric resonance, the transparency peak, and the symmetric higher resonance. Red arrows: magnetic dipoles; Blue arrows: electric dipoles.

Figure S2 shows the equivalent dipole model of (a-c) bright-dark SRRs and (d-f) bright-dark-dark-bright SRRs for three resonant response: the lower asymmetric resonance, the transparency peak, and the symmetric higher resonance. The electric and magnetic dipoles in the different directions will suppress each other. As the mirror-like symmetrical arrangement, the radiation behavior of bright-dark-dark-bright SRRs is rather like an electric dipole in -y-direction.
